# Analysis of the Effects of Microwave Combined Induction Heating on Steamed Pork with Rice Powder

**DOI:** 10.3390/foods13132026

**Published:** 2024-06-26

**Authors:** Su-Der Chen, Chuang-Hsing Kuo, Rong-Shinn Lin

**Affiliations:** 1 Department of Food Science, National Ilan University, Number 1, Section 1, Shen-Lung Road, Yian City 260007, Yilan County, Taiwan; sam861118kuo@gmail.com; 2 Department of Biotechnology and Animal Science, National Ilan University, Yian City 260007, Yilan County, Taiwan

**Keywords:** microwave combined induction heating, steamed pork, lethality, quality

## Abstract

This study investigates the application of microwave combined induction heating (MCIH) to steam ready-to-eat pork with rice powder, emphasizing the advantages of rapid and uniform heating. The experimental setup included a mixture of 180 g pork strips, 30 g rice powder, and 10 g water in a CPET tray using MCIH with 1080 W microwave (MW) and 130 °C induction heating (IH) for 150 s. The results showed a quick temperature increase rate of 0.56 °C/s that achieved pasteurization against a variety of pathogenic bacteria, such as *Listeria monocytogenes*, but not *Clostridium botulinum*, by lethality calculation. Compared to typical electric cooker steaming, MCIH significantly shortened cooking time (8.6 times faster). To address rice starch gelatinization, two-stage heating techniques to steam pork with rice powder were MCIH: 150 s, and then IH: 60 s (MW1), and MCIH: 180 s, and then IH: 30 s (MW2), with no significant differences seen in color or the nine-point taste scale between treatment groups. MCIH groups had smaller shear forces than control. After MCIH cooking, no microbial counts were detected in the MW1 and MW2 groups initially, and the pork with rice powder had a shelf life of 14 days at 4 °C based on aerobic plate count assay.

## 1. Introduction

Ready-to-eat (RTE) food is a product that has been precooked, packaged, and is ready to eat without any additional preparation. Because of its convenience and ease of preparation, the global consumer market for RET food is expanding annually worldwide [1]. Demand for refrigerated RTE meals in retail and delivery is growing. When it comes to product safety and quality, ready-to-eat food needs the use of proper pasteurization procedures to inactive food-borne pathogens and reduce the spoilage microorganisms for extension of the shelf life of food [2,3].

Choosing pasteurization conditions to control potential pathogen contamination in food, considering storage conditions after processing, and general research on RTE meat products indicate that *Listeria monocytogenes* remains active even at temperatures below 0 °C [4,5]. Therefore, *Listeria monocytogenes* is used as an indicator microorganism in refrigerated RTE food. The European Chilled Food Federation recommends reaching 70 °C for 2 min at the coldest point to achieve the 6-log reduction requirement [2,6]. In 2017, the United States Food and Drug Administration (FDA) issued draft guidelines for controlling *Listeria monocytogenes* in RTE food, recommending a decrease of 5 log or more to assure food safety [7].

Microwaves are electromagnetic waves, with frequencies ranging from 300 MHz to 300 GHz. To avoid communication interference, the Federal Communications Commission (FCC) in the United States regulates industrial microwave frequencies at 915 and 2450 MHz [8]. The theory of microwave heating is based on microwave absorption by food, which causes polar molecules to rotate or ions to move due to resonance effects, generating frictional heat and rapidly heating the food. Therefore, microwave heating overcomes the slow heat transfer resistance and rapidly reaches the required sterilizing temperature [3]. Additionally, shorter sterilization times prevent the loss of heat-sensitive nutrients, particularly vitamins B and C, which are susceptible to thermal degradation, retaining the commercial value of the product [9]. A microwave-assisted pasteurization system (MAPS) utilizes microwave as a heat source, employing a four-stage process of preheating, microwave heating, holding, and cooling to achieve commercial sterilization of RTE foods. MAPS provides the advantages of rapid and continuous processing [2]. There has been substantial study on the use of MAPS sterilization systems in RTE foods such as rice and pasta [1,10]. Continuous microwave-assisted thermal sterilization (MATS) systems with throughputs between 30 and 45 meals per minute are currently in operation in India to commercially produce shelf-stable meals [3].

The microwave combined induction heating (MCIH) system or microwave-assisted induction heating (MAIH) system was developed by Bottle Top Machinery Co., Ltd. (Nantou County, Taiwan) [11]. It contains two heating sources—a 2450 MHz microwave in the top part of the machine and an electromagnetic induction heating in the bottom part of the machine—described in detail by Lee et al. [12]. Food is placed in crystallized polyethylene terephthalate (CPET) trays, sealed with polyethylene terephthalate (PET) film by a heat-sealing machine, and then locked into the microwave resonant chamber of the MCIH for cooking and pasteurization. This system, which includes a microwave on the top, can quickly heat food inside the CPET. However, the heat distribution is uneven. Therefore, induction heating on the bottom is used to ensure a more even temperature distribution of the food.

Tsai et al. [13] conducted a study on cooking shrimp using the MAIH system and found that simultaneous activation of microwaves and induction heating resulted in better pasteurization effects than using a single heat source. Other studies have shown that cooking shrimp with a microwave power of 1300 W and induction heating temperature of 130 °C for 90 s achieves rapid sterilization [11]. In terms of heating uniformity, Tsai et al. [14] found fresh clams were heated via MAIH to 130 °C for 110 s and to 90 °C for 130 s, respectively. They found that when heated for 110 s, the overall sample temperature was approximately 100 °C. Thus, MAIH processing can prolong the storage life of the heat-treated clams from 10 days (boiling water for 150 s) to 30 days at 4 °C.

Pork strips mixed with salt koji at a 5:1 ratio using high-pressure processing (HPP) can be tenderized by enzymes in a solution while marinating quickly. With 100 MPa, 10 min treatment, the salt content significantly increased from 0.5% to 0.9%. The control group showed a shear force of 56.12 N, while after HPP treatment, the lowest shear force was 48.73 N, indicating that HPP resulted in both salt curing and tenderizing effects [15].

Steamed pork powder is mainly made from crushed rice or rice flour, mixed with seasonings. The outer layer of pork strips is coated with rice powder and then cooked in an electric cooker for 25 min. This method utilizes external rice powder to absorb pork juices lost during cooking and to gelatinize the rice starch. It requires a longer cooking time and has a shorter shelf life. The rapid heating and uniform heating characteristics of the MCIH system for preparing RTE food present an opportunity. The objective of this study was to develop the MCIH process for steaming pork with rice powder to shorten the heating time, achieve better pasteurization value, and extend the storage period at 4 °C.

## 2. Materials and Methods

### 2.1. Materials

The pork hind leg meat used in this experiment was purchased from Yahsen Frozen Foods Co., Ltd. (Taoyuan City, Taiwan). The rice koji was purchased from Green Life Co., Ltd. (Taipei City, Taiwan). The nutritional information for rice koji per 100 g is as follows: 367 kcal energy, 7.2 g of protein, 0.7 g of fat, 82.9 g of carbohydrates, and 40 mg of sodium. The rice powder was purchased from Zui Fa Food Co. (New Taipei City, Taiwan), with the following nutritional information per 100 g: 310 kcal energy, 6.0 g of protein, 1.0 g of fat, 69.0 g of carbohydrates, and 126 mg of sodium. 3M™ Petrifilm™ Aerobic Count Plates (3M Health Care. Co., St. Paul, MN, USA) were used for microbial analysis.

### 2.2. Experimental Design

**Figure 1:**
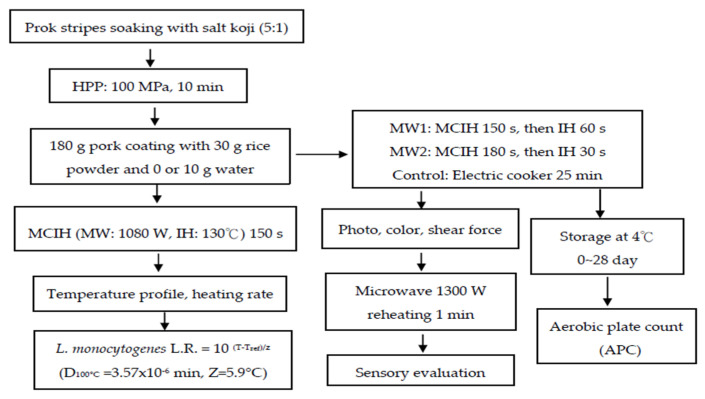
Experimental design of this study.

The experimental design is illustrated in Figure 1. It consists of two parts: the first part involves calculating the lethality of *L. monocytogenes* based on the temperature curve of the MCIH process. The second part focuses on analyzing the quality and sensory evaluation of MCIH-steamed pork with rice powder, followed by microbial analysis during storage at 4 °C.

### 2.3. Salt Koji Preparation

Rice koji was mixed with a 10% salt solution at a ratio of 1:5 (*w*/*w*), and fermented at 25 °C for 3 days. Afterward, it was kept in a refrigerator for later use. The salt content of the salt koji solution dropped from 10% to 2% after 3-day fermentation.

### 2.4. HPP Treatment

The pork was cut into strips of 2 cm × 1 cm × 1 cm. The salt koji solution was mixed with pork strips at a ratio of 5:1 in a bag and vacuum-sealed. Five liters of reverse osmosis (RO) water were filled into the chamber of the HPP equipment (HPP600 MPa/6.2 L, Kuentai International Co., Ltd., Yunlin, Taiwan). The samples were then placed in a pressure chamber, and HPP processing was carried out at 100 MPa and a holding time of 10 min.

### 2.5. Steamed Pork with Rice Powder Preparation

The 180 g pork strips treated with HPP were removed from the vacuum bag and placed in crystallized polyethylene terephthalate (CPET) trays (inner diameter 6.5 cm × height 3.0 cm, Bottle Top Machinery Co., Ltd., Nantou County, Taiwan). Then, 30 g of rice powder was added and the mixture was combined to coat the pork strips evenly. To improve the starch gelatinization effect of the rice powder, the surface was sprayed with 0 or 10 g water. Subsequently, cooking was then carried out with either an electric cooker or MCIH system.

### 2.6. MCIH Cooking Process

The pork strips were coated with rice powder and placed in CPET trays before being packaged in a heat-sealing machine at 160 °C. Following sealing, the CPET trays were placed in the MCIH container and locked tightly. The chamber was then put into the MCIH equipment for cooking and pasteurization under 1080 W microwave and 130 °C induction heating for 150 s.

To improve rice gelatinization, a two-stage heating procedure was carried out, as follows. (1) MW1: 1080 W microwave and 130 °C induction heating for 150 s, followed only by induction heating, which was separately turned on for 0, 30, and 60 s. (2) MW2: 1080 W microwave and 130 °C induction heating for 180 s, followed by only induction heating for 0, 30, and 60 s. After heating under the previously mentioned MCIH conditions, the samples were immediately removed from the MCIH container.

For the control group, cooking was carried out using an electric cooker (TAC-10L-DGU, Tatung, Taipei, Taiwan). In this method, 180 g water was added to the outer pot, and cooking was completed after 25 min of steaming.

The flow of preparing and processing pork with rice power was: cut pork into strips → mix pork with salt koji solution ((5:1) → vacuum-seal bag → place samples in HPP chamber (100 MPa, 10 min) → mix 180 g pork and 30 g rice powder w/or w/o 10 g water → place in a CPET tray → heat-seal tray → 25 min steaming in an electric cooker as control or cooking by MCIH (1080 W MW, 130 °C IH heating, 150 s or 180 s or extending IH heating to 30 s or 60 s).

### 2.7. A Wireless Temperature Detector Used to Plot the Heating and Lethality Curves

The operation involved a wireless temperature detector with microwave heating protection (PVQ/1Tc/3_1.9C30B, TMI-USA, Inc., Reston, VA, USA), inserting the center of pork strips and measuring temperature during MCIH heating. To use the wireless temperature detector, we first installed Qlever software (QLEVER, TMI-USA, Inc., Reston, VA, USA) and set it to record every 10 s for a total duration of 15 min. After MCIH heating was finished, the wireless temperature detector was removed from the pork strips and converted into Excel format to record time and temperature data for plotting the heating curve.

The following D and Z values of *Listeria monocytogenes* are utilized in the lethal rate (*L.R*.) formula of $L.R.=10^{\left(\frac{T-T_{ref}}{Z}\right)}$, due to it serving as a key indicator microorganism in refrigerated RTE meat products. The lethality (L) was accumulated using numerical integration, where L = $\int_{0}^{t}f\left(x\right)=\left(\frac{h}{2}\right)(f_{0}+2f_{1}+2f_{2}+\cdots +2f_{n-1}+2f_{n})$, with h being 10/60 min, and *f* representing the lethality rate (*L.R*.) at each time point [16]. Finally, the L/D_100 °C_ for different pathogens was calculated to determine the pasteurization effect.

### 2.8. Color Analysis

The color of the samples was measured with a color difference meter (Hunter LAB, Color Flex, Virginia, VA, USA) and standardized against a white calibration plate (X = 82.48, Y = 84.23, Z = 99.61, L* = 92.93, a* = −1.26, b* = 1.17). The parameters determined were the degree of lightness (L*), redness (+a*), greenness (−a*), yellowness (+b*), and blueness (−b*) [17]. All experiments were performed in six repetitions.

### 2.9. Shear Force of Products

The shear force was measured using a texture analyzer (TA.XT plus, Stable Micro System, Surrey, UK), with samples cut using a Meullenet-Owens Razor shear blade (Stable Micro System, Surrey, UK). The testing speed was set to 2 mm/s, and the testing distance was 20 mm. Samples (2 cm × 1 cm × 1 cm) were measured at 25 °C. Each treatment was measured for shear force five times, and the average value was taken as the shear force.

### 2.10. Microbial Analysis under 4 °C Storage

The RTE in-container products were stored at 4 °C for 0, 7, 14, 21, and 28 days for microbial analysis. For aerobic plate count (APC), each 10 g sample was placed in a sterilized bag and then 90 mL of sterile water was added, and the mixture was homogenized for 1 min using a stomacher machine. Subsequently, 1 mL of the supernatant was taken for microbial dilution cultivation. This represented a 10-fold dilution. Further dilutions were made as required based on the desired dilution factor. The samples were then inoculated onto 3M Petrifilm™ and incubated at 37 °C incubator (LM-600R, Yihder Technology Co., Ltd., Taipei, Taiwan) for 48 h. The number of colonies on the culture was calculated and then converted to aerobic plate count.

### 2.11. Sensory Evaluation

Steamed pork with rice powder stored in a refrigerator at 4 °C was reheated in a microwave oven (1300 W) for 1 min. Then, a nine-point hedonic sensory evaluation of the products was evaluated by 44 inexperienced panelists on attributes including color appearance, aroma, juiciness, tenderness, and overall acceptability. Ratings were given on a nine-point scale (1 = extremely dislike, 9 = extremely like), with higher values indicating stronger preference. The results were determined by averaging the scores given by each panelist.

### 2.12. Statistical Analysis

The experimental results are expressed as means ± standard deviation. The USA Statistical Analysis System (SAS 9.4, SAS Institute, Cary, NC, USA) was used for data analysis. One-way analysis of variance (ANOVA) and Duncan’s multiple range test were employed for significant difference analysis, comparing the means of each group at a significance level of 5% (*p* < 0.05).

## 3. Results and Discussion

### 3.1. Influence of MCIH Heating Process on the Heating Rate of Steamed Pork with Rice Powder

**Figure 2:**
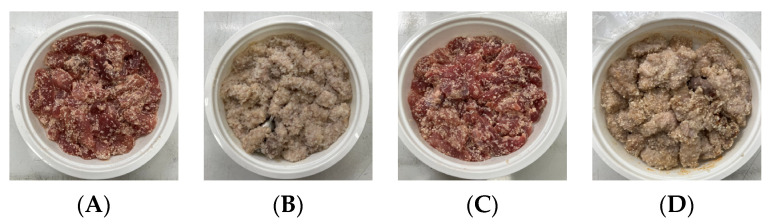
Photos of steamed pork with rice powder (pork: 180 g, powder: 30 g) in an electric cooker (steam 25 min: (**A**) raw, (**B**) cooked), and MCIH heating 150 s (MW 1080 W, IH:130 °C): (**C**) raw, (**D**) cooked).

The conventional method of cooking steamed pork with rice powder was coating the pork strips with rice powder and then steaming them in an electric cooker. The rice powder is primarily used to season the product and absorb meat juices. Figure 2 shows photos of steamed pork with rice powder cooked with an electric cooker and MCIH (MW: 1080 W, IH: 130 °C). The gelatinization of the rice powder differed significantly across the two cooking methods. The rice starch was fully gelatinized and appeared more uniform when cooked in an electric cooker, because the longer cooking time allowed it to absorb more water, and moisture is a crucial factor in starch gelatinization [18] (Figure 2B). However, white rice starch particles were visible using MCIH cooking, which could be related to insufficient water and a short absorption time (Figure 2D). Recording the weight change during electric cooker cooking revealed that the pork strips with rice powder absorbed approximately 8–10 g of water. Therefore, the formula should be 180 g pork strips and 30 g rice powder with an extra 10 g of water sprayed to increase the heating rate and improve rice starch gelatinization.

**Figure 3:**
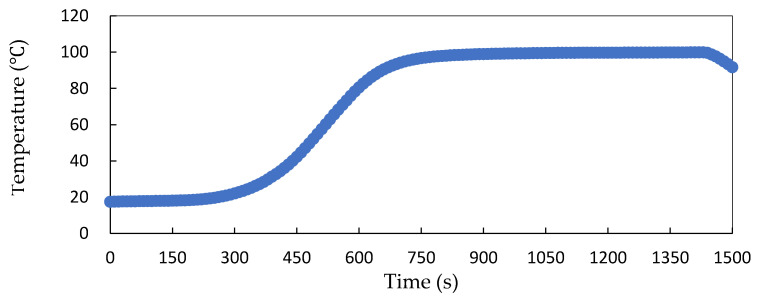
Heating curves of traditional electric cooker cooking steamed pork with rice powder (pork: 180 g, powder: 30 g).

**Figure 4:**
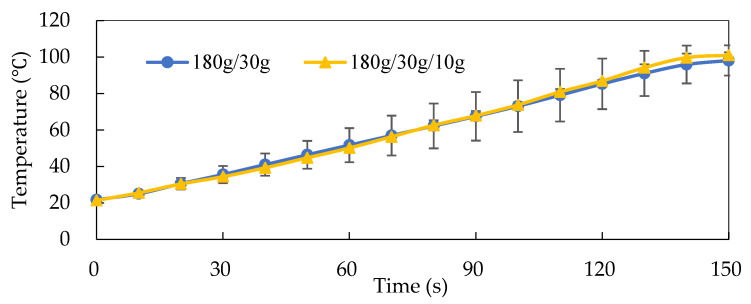
Heating curves of different formulae for steamed pork (pork: 180 g, powder: 30 g, water: 0 or 10 g) during MCIH (MW:1080W, IH: 130 °C) 150 s heating.

Figure 3 shows the heating curve of traditional steamed pork with rice powder cooked in an electric cooker (pork strips: 180 g, rice powder: 30 g). It took until 600 s for the center temperature to reach 80.5 °C. Figure 4 shows the heating curves of steamed pork strips with various formulations (pork strips: 180 g, rice powder: 30 g, and the addition of RO water: 0 or 10 g) heated by MCIH (MW: 1080 W, IH: 130 °C) for 150 s. The results indicated that the composition of pork strips in the CPET tray affected the heating rate of steamed pork with rice powder. In the group without addition of 10 g RO water to 180 g of pork strips with 30 g rice powder, the center temperature reached 85.32 °C after 120 s of cooking. When the temperatures at various points were fitted into the linear regression equation, y = 0.5343x + 20.023, R^2^ = 0.9986, where y represented temperature and x represented time, the heating rate was 0.53 °C/s. In addition, the group that had 10 g of water added took 120 s to reach a temperature of 86.92 °C. When fitted into the linear regression equation, y = 0.5631x + 18.357, R^2^ = 0.9958, the heating rate was 0.56 °C/s (Table 1). Moreover, MCIH shortens the cooking time by about six times compared to electric cooker steaming, and the rapid heating characteristic of MCIH is also reflected in the pasteurization effect.

The heating mechanism of microwave-heated food is influenced by the dielectric loss factor (ε″), which represents the ability to convert absorbed electrical energy into heat energy. Heating conditions vary depending on the composition of the food [19]. Lyng et al. [20] found that adding 25% water increases the dielectric constant (ε′) and dielectric loss factor (ε″) of ground beef. Bengtsson and Risman [21] found a positive correlation between moisture content and dielectric constant (ε′) in beef. Therefore, adding 10 g water to the formula enhanced the heating rate of steamed pork with rice powder during MCIH cooking.

In addition, this study used a pre-treatment process for the raw material pork trips, utilizing HPP with a salt koji marinade, which enhanced the salinity level from 0.50% to 0.76%. This alteration also increased the dielectric properties of foods, resulting in a faster heating rate of MCIH heating. Al-Holy et al. [22] found that the salinity of sturgeon caviar increased during marination, resulting in an increasing dielectric loss factor (ε″) from 40.5 to 73.6 at 915 MHz microwave. Furthermore, adding 3% salt to beef burgers might increase their dielectric loss factor, increasing the temperature rate of the burger bun during microwave reheating [23].

### 3.2. Effect of MCIH Heating on Pasteurization of Steamed Pork with Rice Powder

Meat products high in nutrients such as proteins and vitamins may be susceptible to microbial contamination and spoilage. The D_100 °C_ and Z values of *Listeria monocytogenes* were reported as 3.57 × 10^−6^ min and 5.9 °C, respectively [24]. These values are utilized in the lethal rate (*L.R.*) formula, $L.R.=10^{\left(\frac{T-T_{ref}}{Z}\right)}$, and the overall lethality was then summed using numerical integration [16]. The MCIH system was used to cook 180 g pork with 10 g rice powder and 10 g water (Product II). The lethality of *Listeria monocytogenes* was 0.29 min, resulting in 8.14 × 10^4^ D_100 °C_ of a pasteurization value (Table 2) after 150 s heating from the temperature profile (Figure 4).

Table 3 shows the D and Z values and lethality of common pathogens (including *Clostridium botulinum*, *Escherichia coli* O157:117, *Salmonella typhi*, *Campylobacter jejuni*, and *Listeria monocytogenes*). These values were crucial for assessing the pasteurization efficiency, which was calculated from temperature profiles (Figure 4) of MCIH (MW: 1080 W, IH: 130 °C) heating steamed pork for 150 s (Product I, consisting of 180 g pork strips and 30 g rice powder, and Product II, consisting of 180 g pork strips, 30 g rice powder, and 10 g water). The D_100 °C_ for the heat-resistant *Clostridium botulinum* is 26.4 min, while for other pathogenic bacteria, it ranges from 3.04 × 10^−8^ to 7.40 × 10^−4^ min. The Z values for these pathogenic bacteria vary from 4.94 to 10.1 °C.

The lethality ranges for Product I and Product II of the steamed pork varied between 0.07–0.15 min and 0.29–0.32 min, respectively. Product II contained an additional 10 g of water and exhibited a notably faster temperature rise rate during MCIH heating for 150 s (Table 3). When considering *Clostridium botulinum*, the pasteurization effect of Product II was assessed by its lethality of 0.32 min dividing D_100 °C_ value of 26.4 min, resulting in 8.72 × 10^−3^, which was insufficient to meet the requirement for a 6-log D reduction in *Clostridium botulinum*. Consequently, steamed pork products with rice powder cooked for 150 s via MCIH must be stored under refrigeration. However, for other pathogenic bacteria, the pasteurization efficiency was high (Table 3).

Auksornsri and Songsermpong [1] explored the sterilization of RTE rice using MAPS in their study. The results showed that achieving a 5-log reduction in pasteurization using traditional heat sources (such as hot water) required a total of 420 s. Subsequent storage at 4 °C for 30 days showed no growth of *Listeria monocytogenes*. In another investigation focusing on the pasteurization of RTE foods utilizing the MCIH system, Tsai et al. [29] investigated the cooking and pasteurization of clams using an 1180 W microwave with induction temperatures set at 90 °C and 130 °C. The results indicated that heating at both induction temperatures of 90 °C for 110 s and 130 °C for 100 s led to the absence of total counts, psychrophilic bacteria, and *Escherichia coli*. Similarly, MCIH heating of shrimp using a 1080 W microwave and induction temperature set at 130 °C for 90 s or at 90 °C for 120 s exhibited effective pasteurization, with no detection of total counts, psychrophilic bacteria, or *Escherichia coli* [12].

Comparing the sterilization effects of a traditional electric cooker and MCIH (MW: 1080 W, IH: 130 °C) in cooking steamed pork with rice powder (pork strips: 180 g, rice powder: 30 g) against *Listeria monocytogenes*, the findings revealed significant differences. The traditional electric cooker required 1290 s to achieve approximately a 5.9-log reduction in pasteurization, while MCIH only required 150 s, a time reduction of nearly 8.6-fold. These outcomes are attributed to heat transfer mechanisms. When food contains polar water molecules or ions, MCIH induces resonance effects, leading to volumetric heating and faster heat distribution than the traditional electric cooker, which primarily relies on conduction and convection [1,2]. Therefore, MCIH exhibits promising potential for advancing the preparation and pasteurization of RTE foods.

### 3.3. MCIH Cooking Processing and Quality Analysis for Steamed Pork with Rice Powder

Table 4 shows photos of steamed pork with rice powder (pork strips: 180 g, rice powder: 30 g, water: 10 g) heated by MCIH (MW: 1080 W, IH: 130 °C) for 150 s and 180 s, followed by individual induction heating for 0, 30, and 60 s. In the MCIH heating for the 150 s group, gelatinization of rice granules was completed after 60 s of induction heating, while in the MCIH + IH heating for the 180 s group, this was accomplished after 30 s of induction heating. Subsequently, the quality analysis compared two different MCIH cooking conditions—MCIH: 150 s, and then IH: 60 s (MW1) and MCIH: 180 s, and then IH: 30 s (MW2)—along with traditional electric cooker heating methods.

Table 5 shows photos and color of steamed pork with rice powder cooked using three methods: control (electric cooker), MW1 and MW2. The L* values were 55.72, 58.67, and 55.66, respectively. Notably, MW1 displayed a significant difference from the other two groups, probably due to its shorter microwave heating time. The a* values were 1.03, 0.86, and 1.04, respectively, with no significant differences among the three groups. The b* values were 9.34, 10.59, and 9.92, respectively, with MW1 showing a slightly larger b* value than the control group.

However, this contrasts with the trends observed in the study of MCIH-cooked shrimp by Hwang et al. [30]. Their research using MCIH heating (1180 W microwave power, induction temperature 130 °C) revealed that increasing the microwave heating time from 100 s to 120 s significantly increased the L* value from 52.65 to 58.09. This increase in lightness was attributed to the reduction in pigment activity and protein denaturation, which enhanced light reflection of the shrimp surface and resulted in a white appearance [30]. This difference could be attributed to variations in the types of pork and seafood products. Furthermore, research on microwave-cooked beef slices showed a significant decrease in L* value, from 47.75 to 27.39, due to protein denature, when the microwave heating time was increased from 2.5 min to 10 min [31], which was similar to the trends observed in this study. In general, cooking food at high temperatures for a long time can cause a Maillard reaction, resulting in a drop in the L* value, which measures lightness. However, if food is heated at high temperatures for a short time, the L* value may remain high.

The shear forces for the control, MW1, and MW2 were 67.78 N, 47.49 N and 52.74 N, respectively (Table 5). Both groups cooked using the MCIH method (MW1 and MW2) exhibited significantly lower values than the control group. This indicated that steamed pork with rice powder cooked using MCIH was more tender than the control, likely due to the prolonged cooking time using the electric cooker.

Table 6 shows the nine-point hedonic sensory evaluation of steamed pork with rice powder cooked by MCIH and the control group (cooked in an electric cooker). There were 44 evaluators, and no significant differences were found in any of the scoring items, indicating that steamed pork with rice powder cooked by MCIH and the control group (cooked in an electric cooker) was similar. Therefore, the formulation and process of developing steamed pork with rice powder using MCIH achieved sensory evaluation levels comparable to those of steamed pork with rice powder cooked in an electric cooker.

The results were consistent with trends observed in other MCIH heating studies. Lee et al. [12] conducted a nine-point hedonic evaluation of shrimp cooked using MCIH. Results showed no significant differences in color, smell, texture, mouthfeel, or overall acceptance between groups cooked at an induction temperature setting of 90 s for 100 s or 80 s compared to a control group. Similarly, in other studies on microwave-heated RTE foods, such as a study on the use of MAPS for sterilizing instant rice, significant differences were observed compared to the control group (hot water) [1].

Finally, microbial testing was performed on the rice powder, revealing a count of 2.88 log CFU/g. Table 7 shows the results of the microbial count stability test of steamed pork with rice powder cooked by MCIH (MW: 1080 W, IH: 130 °C) and stored at 4 °C. After cooking, no microbial counts were found in the MW1 and MW2 groups. However, on the seventh day of storage, the microbial counts were 2.28 and 1.77 log CFU/g, respectively, and by the 14th day, they had 3.51 and 3.28 log CFU/g respectively. By the 21st day, the microbial counts had exceeded 105. However, Kuo et al. [15] found no microbial counts in their experiment on MCIH-cooked pork strips stored at 4 °C for up to 28 days. Therefore, the variation in results could be attributed to the microbial count of the rice powder itself, resulting in a shelf life of only 14 days for steamed pork with rice powder cooked by MCIH (pork: 180 g, powder: 30 g, water: 10 g) under refrigeration.

## 4. Conclusions

The formulation for steamed pork with rice powder consisted of 180 g pork strips and 30 g rice powder, with an additional 10 g of water sprayed on the surface to enhance rice starch gelatinization. The mixture was cooked using MCIH with a 1080 W microwave and 130 °C induction heating for 150 s or extended IH heating for 60 s, instead of the traditional 25 min steaming in an electric cooker. The color and sensory evaluation of the MCIH and control groups were similar, except MCIH had a more tender structure. This MCIH in-packaged RTE product achieved a pasteurization value with more than a 6-log D reduction for *Listeria monocytogenes.* After MCIH cooking, no microbial counts were initially detected, and the shelf life was extended to 14 days when stored at 4 °C.

## Figures and Tables

**Table 1:** The heating rate of steamed pork products by MCIH (MW:1080 W, IH:130 °C) heating for 150 s.

Formula	Pork (g)/Rice Powder (g)/Water (g)	
	180/30/0	180/30/10
Linear regression	y = 0.5343x + 20.023	y = 0.5631x + 18.357
R^2^	0.9986	0.9958
Heating rate (°C/s)	0.53	0.56

y is temperature (°C) and x is time (s).

**Table 2:** Temperature profile and lethality of *Listeria monocytogenes* (D_100 °C_ = 3.57 × 10^−6^ min, Z = 5.9 °C) during MCIH (MW:1080 W, IH:130 °C) heating of steamed pork products (pork: 180 g, powder: 30 g, water: 10 g) for 150 s.

Time (s)	Temperature (°C)	Lethal Rate	Lethality (min)	L/D_100 °C_
0	21.39	4.75 × 10^−14^	0.00	0.00
10	25.53	2.39 × 10^−13^	2.39 × 10^−14^	6.68 × 10^−9^
20	30.42	1.61 × 10^−12^	1.78 × 10^−13^	4.98 × 10^−8^
30	34.42	7.67 × 10^−12^	9.51 × 10^−13^	2.66 × 10^−7^
40	39.27	5.09 × 10^−11^	5.83 × 10^−12^	1.63 × 10^−6^
50	44.76	4.34 × 10-^10^	4.62 × 10^−11^	1.29 × 10^−5^
60	50.17	3.58 × 10^−9^	3.81 × 10^−10^	1.07 × 10^−4^
70	56.33	3.97 × 10^−8^	3.98 × 10^−9^	1.12 × 10^−3^
80	62.64	4.65 × 10^−7^	4.61 × 10^−8^	1.29 × 10^−2^
90	67.87	3.58 × 10^−6^	3.83 × 10^−7^	1.07 × 10^−1^
100	73.81	3.64 × 10^−5^	3.71 × 10^−6^	1.04 × 10^0^
110	80.94	5.88 × 10^−4^	5.58 × 10^−5^	1.56 × 10^1^
120	86.92	6.07 × 10^−3^	6.10 × 10^−4^	1.71 × 10^2^
130	94.21	1.04 × 10^−1^	9.81 × 10^−3^	2.75 × 10^3^
140	99.71	8.93 × 10^−1^	9.29 × 10^−2^	2.60 × 10^4^
150	101	1.48 × 10^0^	2.90 × 10^−1^	8.14 × 10^4^

**Table 3:** Lethality of pathogenic bacteria during MCIH (MW:1080 W, IH:130 °C) heating of steamed pork products for 150 s.

Pathogenic Bacteria	D_100 °C_ (min)	Z (°C)	Reference	Pork (g)/Powder (g)/Water (g)	
				180/30/0	180/30/10
				Lethality (min)	
*Clostridium botulinum*	26.4	10	[25]	0.15	0.32
*Escherichia coli* O157	2.27 × 10^−5^	7.39	[26]	0.11	0.30
*Salmonella typhi*	7.40 × 10^−4^	10.1	[27]	0.15	0.32
*Campylobacter jejun* *i*	3.04 × 10^−8^	4.94	[28]	0.07	0.29
*Listeria monocytogenes*	3.57 × 10^−6^	5.90	[24]	0.09	0.29

**Table 4:** Photos of steamed pork with rice powder heated by MCIH (MW: 1080 W, IH: 130 °C) for 150 s and 180 s, then heated by induction heating for 0, 30, and 60 s.

IH Holding (s)	0	30	60
MCIH heating 150 s	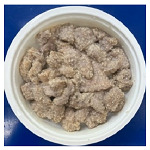	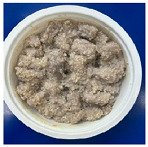	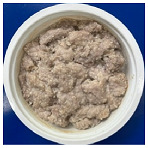
MCIH heating 180 s	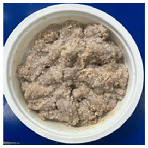	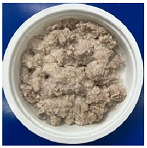	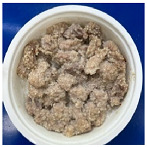

**Table 5:** Photos, shear force, and color of steamed pork with rice powder by MCIH cooking group and control group.

Color	Photo	L*	a*	b*	Shear Force (N)
Control (electric cooker 25 min)	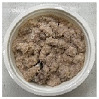	55.72 ± 1.98 ^b^	1.03 ± 0.36 ^a^	9.34 ± 0.96 ^b^	67.78 ± 7.82 ^a^
MW1	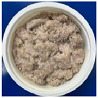	58.67 ± 1.82 ^a^	0.86 ± 0.52 ^a^	10.59 ± 1.30 ^a^	47.49 ± 4.12 ^b^
MW2	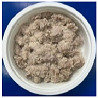	55.66 ± 1.46 ^b^	1.04 ± 0.36 ^a^	9.92 ± 0.92 ^ab^	52.74 ± 6.83 ^b^

MW1: MCIH (MW:1080 W, IH: 130 °C) 150 s, and then IH: 60 s; MW2: MCIH (MW:1080 W, IH: 130 °C) 180 s, and then IH: 30 s. ^a,b^ Means with different superscript letters (*n* = 6) in the same column are significantly different (*p* < 0.05).

**Table 6:** Sensory evaluation of steamed pork with rice powder using MCIH and control.

Item	Control	MW1	MW2
Appearance	6.25 ± 1.37 ^a^	6.02 ± 1.45 ^a^	5.89 ± 1.40 ^a^
Smell	6.20 ± 1.39 ^a^	5.77 ± 1.49 ^a^	6.34 ± 1.57 ^a^
Juiciness	5.93 ± 1.72 ^a^	5.84 ± 1.84 ^a^	5.93 ± 1.89 ^a^
Tenderness	5.91 ± 1.70 ^a^	6.30 ± 1.92 ^a^	5.70 ± 1.89 ^a^
Overall acceptance	6.36 ± 1.42 ^a^	6.43 ± 1.59 ^a^	6.20 ± 1.58 ^a^

Control: steam in an electric cooker for 25 min. MW1: MCIH: 150 s, IH: 60 s; MW2: MCIH 180 s, IH: 30 s. Means with different superscript letters (*n* = 44) in the same column are significantly different (*p* < 0.05).

**Table 7:** Total bacterial count of steamed pork with rice powder by MCIH cooking.

Storage (Day)	MW1 log CFU/g	MW2 log CFU/g
0	N.D.	N.D.
7	2.28 ± 0.09	1.77 ± 0.07
14	3.51 ± 0.04	3.28 ± 0.15
21	5.40 ± 0.01	5.30 ± 0.08
28	6.16 ± 0.02	6.41 ± 0.06

MW1: MCIH: 150 s, IH: 60 s; MW2: MCIH 180 s, IH: 30 s (*n* = 3). N.D. is an abbreviation of Not Detected. The initial total plate count of rice powder is 2.88 log CFU/g.

## Data Availability

The data presented in this study are available on request from the corresponding author. The data are not publicly available, due to privacy and ethical reasons.
